# Quality function deployment modified for the food industry: An example of a granola bar

**DOI:** 10.1002/fsn3.1014

**Published:** 2019-04-03

**Authors:** Tzu‐Yun Wang, Hsin‐I Hsiao, Wen‐Chieh Sung

**Affiliations:** ^1^ Department of Food Science National Taiwan Ocean University Keelung Taiwan; ^2^ Institute of Food Safety and Risk Management National Taiwan Ocean University Keelung Taiwan

**Keywords:** fuzzy QFD, granola bar, house of quality, NDP, quality function deployment

## Abstract

Applying quality function deployment (QFD) in the food industry is complex. The objective of this study was to provide a modified QFD approach to facilitate its use and test it on a granola bar. A 7‐step model is proposed (modified from the 9‐step sensory attributes model) incorporating competitive analysis to acquire final importance ratings of customer needs, the application of fuzzy logic in surveys and interviews, and replacing precise value of relative technical ratings by priority rankings. The 7‐step model starts with understanding customer needs (WHATs) and prioritizing their importance based on the customer survey and competitive analysis, followed by identification of product's sensory attributes (HOWs) and the strength of their relationship with customer needs. Lastly, relative technical ratings—to develop priority rankings for the product development team—are determined based on the degree of importance of the WHATs and the strength of the relationships between the WHATs and the HOWs. Testing the 7‐step model on granola bars showed that the relative technical ratings reflected the majority of customer needs and the importance they attached to those needs.

## 1 INTRODUCTION

The failure rate of new food product launches is high; nearly 50% of new products are removed from the market within a year of their launch (Dijksterhuis, 2016). To keep food products competitive and secure their longevity in the marketplace, time to market (Benner, Linnemann, Jongen, & Folstar, 2003b), continual development based on customer needs (Jacobsen et al.., 2014), internal business communication (Jacobsen et al., 2014), and quality (Dijksterhuis, 2016) are crucial.

Quality function deployment (QFD) is a product development tool based on customer needs (Benner, Linnemann, et al., 2003b) and has the benefits of reaching a compromise between customer needs and production techniques (Chien & Su, 2003), decreasing production costs, and reducing production time (Dursun & Karsak, 2013). Accordingly, it is a tool that well responds to the needs of new product development in the food industry. The four‐phase model is often used, where customer demands are deployed through four matrices to production planning (Hauser & Clausing, 1988). Among the four matrices, the house of quality (HoQ), the first matrix, whereby customer‐driven technical measures are the main outputs, is most well known and widely practiced. Quality function deployment has been implemented in the food industry since 1987 (Costa, Dekker, & Jongen, 2000), but its application poses some problems. For example, it is hard to determine precise values for technical measures, since these are influenced by one another in food products (Benner, Linnemann, et al., 2003b). It is complex to practice and should be adjusted to a more hands‐on tool (De Pelsmaeker, Gellynck, Delbaere, Declercq, & Dewettinck, 2015). Moreover, the QFD results are too subjective and depend to a great extent on the people who use it (De Pelsmaeker et al., 2015). Chan and Wu (2005) presented a systematic and operational approach to quality function deployment (QFD) and proposed a 9‐step model (step 1: Identify customers & collect customer needs/WHATs, step 2: Determine relative importance ratings of WHATs, step 3: Identify competitors, conduct customer competitive analysis & set customer performance goals for WHATs, step 4: Determine final importance ratings of WHATs, step 5: Generate technical measures (HOWs), step 6: Determine relative between WHATs and HOWs, step 7: Determine initial technical ratings of HOWs, step 8: Conduct technical competitive analysis & set technical performance goals for HOWS, step 9: Determine final technical ratings of HOWs). Bech, Hansen, and Wienberg (1997) suggested measuring the sensory attributes by descriptive sensory analysis. This modified sensory approach was applied in areas, such as kiwi peel product development (Vatthanakul, Jangchud, Jangchud, Therdthai, & Wilkinson, 2010), evaluation of likeness of extra virgin oil (Bevilacqua, Ciarapica, & Marchetti, 2012), and improvement of chocolate product (De Pelsmaeker et al., 2015). Therefore, this research aims to develop a simpler QFD approach by modifying the 9‐step model by Chan and Wu (2005) and combining the sensory modified model by Bech et al. (1997) to tackle current problems and evaluate its feasibility by testing on a granola bar.

## 2 RESEARCH METHODS

A 7‐step model was developed on the basis of the 9‐step model by Chan and Wu (2005) and the sensory attributes by Bech et al. (1997). The modifications consisted of incorporating competitive analysis (CA) to acquire final importance ratings of customer needs, CAs weight, applying fuzzy logic (Zadeh, 1965) in surveys and interviews, and replacing the value of relative technical ratings, engineering characteristics by priority rankings.

This study used a granola bar as an example to demonstrate the 7‐step model. The major ingredients of the granola bar contained oats, nuts, grains (sesame and pumpkin seeds), and dried fruit. The granola bar was made by a Taiwanese manufacturer. Three different brands of granola bar manufactured in foreign countries were chosen as competitors based on sales ranking. Only female customers were chosen because granola bars are mainly consumed by female consumer in Taiwan. The detailed 7‐step research method is described below.

### 2.1 Step 1: Identify customers and customer needs (WHATs)

The objective of this step is to identify the product's target customers and their needs. Target customers were determined on the basis of product positioning and market research, and customer needs were acquired through interview (Chan & Wu, 2005).

#### 2.1.1 Interview

According to Griffin and Hauser (1993), 20–30 interviewees are necessary to capture 90%–95% of customer needs. Twenty‐five female customers aged between 20 and 40 were recruited via snowball sampling (Goodman, 1961) via classmates, colleagues, and friends, and were interviewed face‐to‐face. A semistructured interview was used. The interview was divided into two parts: a respondent profile and questions about preferences regarding a granola bar (Bevilacqua et al., 2012) (Supporting Information Appendix S1). The questions were pretested before the interviews were carried out. The interviews were conducted during November and December 2016.

### 2.2 Step 2: Determine the relative importance ratings of customer needs (WHATs)

#### 2.2.1 Survey

A survey approach was used to collect the relative importance rating of customer needs. The questionnaire (see Supporting Information Appendix S2) was divided into two parts, namely a respondent profile and a customer needs rating (Chan & Wu, 2005). The questionnaire was distributed via snowball sampling (Goodman, 1961) through classmates, colleagues, and friends. Respondents were asked to evaluate each customer need in given linguistic importance variables, which were very low (VL), low (L), moderate (M), high (H), and very high (VH). The survey questionnaire was pretested and distributed from January to March 2017.

#### 2.2.2 Analysis

After the importance scores were collected, the relative importance ratings were computed using the following equation (Chan & Wu, 2005):

(1) $$\begin{array}{c} \text{Relative importance ratings}\;\alpha_{m}=\left(\alpha_{m1}+\alpha_{m2}+\cdots +\alpha_{\mathrm{mK}}\right)/K\\ =\sum_{K=1}^{K}\alpha_{\mathrm{mk}}/K,\;m=1,2,\ldots ,M. \end{array}$$

with *m* = number of customer needs, *K* = number of customers that gave scores.

### 2.3 Step 3: Identify competitors and conduct customer competitive analysis

#### 2.3.1 Survey

A survey approach was used to identify competitors and conduct customer competitive analysis. The questionnaire (see Supporting Information Appendix S3) was divided into two parts, consisting of a respondent profile and competitive evaluation of four brands (anonymous) (Chan & Wu, 2005). The questionnaire was distributed via snowball sampling (Goodman, 1961) through classmates, colleagues, and friends. Overall, the responses of 27 female tasters aged 20–40 were collected. Respondents were asked to evaluate in given linguistic performance variables, which were very poor (VP), poor (P), neutral (N), good (G), and very good (VG) after tasting the samples. The survey questionnaire was pretested and distributed during April 2017.

#### 2.3.2 Analysis

The degree of improvement of our own granola bar was computed using the following equation (Chan & Wu, 2005):

(2) $$\text{Improvement ratio}\;\gamma_{m}=g_{m}/x_{m1}$$

with m = number of customer needs (WHATs), *g_m_* = goal for each customer need, and *x_m_*_1_ = performance of own product on each customer need.

The degree of competitiveness was computed using the following equation (Chan & Wu, 2005):

(3) $$\text{Competitive priority ratings}\;\;\beta \left(W_{m}\right)=-\emptyset_{l}\sum p_{\mathrm{ml}}\ln\left(p_{\mathrm{ml}}\right)$$

with m = number of customer needs (WHATs), *l* = number of product, and *p_ml_* = probability distribution of each customer need.

### 2.4 Step 4: Determine the final importance ratings of customer needs

The objective of this step is to re‐prioritize the importance of customer needs based on the degree of competitiveness and improvement.

#### 2.4.1 Analysis

The final importance rating was computed using the following equation (Chan & Wu, 2005):

(4) $$\delta_{m}=\alpha_{m}\times \beta_{m}\times \gamma_{m^{\prime }}\;m=1,2,\ldots ,M.$$

with m = number of customer needs (WHATs).

### 2.5 Step 5: Generate technical attributes and sensory attributes (HOWs)

The technical and sensory attributes related to customer needs were generated by consulting academic experts and a literature review (Bevilacqua et al., 2012).

### 2.6 Step 6: Determine the strengths of the relationships between the WHATs and HOWs

#### 2.6.1 Survey

Three food technicians were invited and surveyed by mail. The survey questionnaire (Supporting Information Appendix S4) contained the relation rating chart, to access the strength of the relationship between each customer need (WHATs) and each technical and sensory attribute (HOWs) according to crisp numbers and fuzzy sets (Chan & Wu, 2005). The relation scores between each WHAT and HOW were given linguistic relation variables, which were very weak (VW), weak (W), moderate (M), strong (G), and very strong (VS). The surveys were conducted during May 2017.

#### 2.6.2 Analysis

Relation scores were computed using the following equation:

(5) $$\begin{array}{c} R_{\mathrm{mn}}=\left(R_{mn1}+R_{mn2}+L+R_{\mathrm{mnE}}\right)/E=\sum_{E=1}^{E}R_{\mathrm{mne}}/E,\\ m=1,2,\ldots ,M,\;n=1,2,\ldots ,N. \end{array}$$

with *m* = number of customer needs, *n* = number of technical and sensory attributes, and *E* = number of experts.

### 2.7 Determine the relative technical ratings of the HOWs

#### 2.7.1 Analysis

This is the main output of the HOQ process (Chan & Wu, 2005). The objective of this step is to determine the degree of importance attached to the improvement of each technical and sensory attribute. After obtaining the final customer needs importance ratings (*δ_m_*) and relationship scores between customer needs (WHATs) and technical and sensory attributes (HOWs) (*R_mn_*), the relative technical ratings were computed using the following equation:

(6) $$\text{Relative technical ratings}\;\varepsilon_{n}=\delta_{m}\times R_{\mathrm{mn}}$$

with *m* = number of customer needs, *n* = number of technical and sensory attributes.

## 3 RESULTS

### 3.1 Customers and customer needs (WHATs)

Twenty‐five interviews were conducted. Overall, 4% of customers were aged between 20 and 24, 88% were aged between 25 and 30, and 8% were aged between 35 and 40. The results of interviews were categorized (Table 1). Customers’ expectations to see, smell, and taste granola bars aligned with the ingredients of granola bars, namely oat granule, nut, and dried fruit. In addition, subjects wanted granola bars with a goof bar shape for easy holding and consumption, since most of them used the bars as meal replacement and snacks. The degree of sweetness and crunchiness was mentioned most. The majority of existing granola bars did not meet these expectations.

**Table 1:** Customer needs (WHATs) for granola bar

Appearance	Aroma
*W*_1_: oat color	*W*_6_: grain odor smelt
*W*_2_: visible oat granule	*W*_7_: nut odor smelt
*W*_3_: visible dried fruit	*W*_8_: dried fruit odor smelt
*W*_4_: visible nut	*W*_9_: honey odor smelt
*W*_5_: easy‐to‐eat bar shape	*W*_10_: toasted odor smelt

Flavor	Texture
*W*_11_: grain flavor tasted	*W*_16_: crunchiness
*W*_12_: oat flavor tasted	*W*_17_: edible crunchy oat granule
*W*_13_: slight sweetness tasted	*W*_18_: edible dried fruit
*W*_14_: dried fruit flavor tasted	*W*_19_: edible nut
*W*_15_: nut flavor tasted	

### 3.2 The relative importance ratings of customer needs (WHATs)

One hundred and thirteen surveys were collected. Ninety‐one of them were accepted as valid by excluding males and those who had never eaten a granola bar before. Overall, 11% of customers were aged between 20 and 24, 55% were aged between 25 and 30, 15% were aged between 31 and 34, 7% were aged 35–40, 5% were aged 41–44, and 7% were aged over 45. Crisp (*α_m_*) and fuzzy relative importance ratings (${\tilde{\alpha }}_{m}$) were computed and analyzed (Table 2). Results show that crunchiness (*W*_16_), grain taste (*W*_11_), and edible crunchy oat granule (*W*_17_) were the most important customer needs.

**Table 2:** Relative importance ratings of customer needs (WHATs) for granola bar

WHATs (*W_m_*)	Relative importance ratings^a^	
	Crisp (*α_m_*)	Fuzzy (${\tilde{\alpha }}_{m}$)
*W*_1_	5.6	[4.6, 6.6]
*W*_2_	8.3	[7.3, 9.3]
*W*_3_	8.1	[7.1, 9.1]
*W*_4_	8.3	[7.3, 9.3]
*W*_5_	7.3	[6.3, 8.3]
*W*_6_	8.1	[7.1, 9.1]
*W*_7_	7.7	[6.7, 8.7]
*W*_8_	7.4	[6.4, 8.4]
*W*_9_	7.1	[6.1, 8.2]
*W*_10_	7.5	[6.5, 8.5]
*W*_11_	8.6	[7.6, 9.6]
*W*_12_	8.2	[7.2, 9.2]
*W*_13_	8.1	[7.1, 9.1]
*W*_14_	8.1	[7.1, 9.1]
*W*_15_	8.0	[7.0, 9.0]
*W*_16_	8.8	[7.8, 9.8]
*W*_17_	8.6	[7.6, 9.6]
*W*_18_	8.4	[7.4, 9.4]
*W*_19_	8.4	[7.4, 9.4]

a Mean value of the scores given by 91 customers.

### 3.3 Competitors and customer competitive analysis

Computed results of the competitive priority ratings (*β_m_*), goals (*g_m_*), and improvement ratios (*γ_m_*) for each customer needs (WHATs) are shown in Table 3. Results show that slight sweetness (*W*_13_) had the highest competitive priority ratings, while visible dried fruit (*W*_3_) needed the most improvement.

**Table 3:** Customer competitive analysis of four granola bar brands

WHATs (*W_m_*)	Customer comparison matrix^a^ *X* = [*x_mn_*]_19 × 4_				Competitive priority ratings (*β_m_*)
	*B*_1_	*B*_2_	*B*_3_	*B*_4_	
*W*_1_	6.3	6.2	5.8	6.2	0.0527
*W*_2_	7.5	6.6	7.0	6.1	0.0524
*W*_3_	4.9	4.1	7.9	6.4	0.0481
*W*_4_	6.8	3.1	4.9	6.9	0.0514
*W*_5_	8.0	7.9	8.0	8.1	0.0514
*W*_6_	4.9	4.2	4.3	4.9	0.0537
*W*_7_	4.6	3.2	4.0	5.4	0.0526
*W*_8_	5.2	5.4	5.1	4.4	0.0536
*W*_9_	4.8	4.9	4.5	4.7	0.0537
*W*_10_	4.2	3.7	3.7	4.3	0.0541
*W*_11_	5.9	4.7	4.8	5.8	0.0533
*W*_12_	5.7	5.0	4.8	5.4	0.0536
*W*_13_	5.7	4.0	3.7	4.1	0.0557
*W*_14_	6.3	5.5	6.3	5.0	0.0525
*W*_15_	5.8	2.8	4.2	6.7	0.0511
*W*_16_	5.5	5.8	5.0	4.7	0.0537
*W*_17_	6.2	5.7	5.7	5.4	0.0531
*W*_18_	5.9	4.3	6.2	5.7	0.0516
*W*_19_	6.3	3.4	4.3	7.1	0.0517

a Mean value of the scores given by 27 tasters.

### 3.4 The final importance ratings of customer needs

The crisp (*δ*_m_) and fuzzy final importance ratings (${\tilde{\delta }}_{m}$) were computed by crisp (*α_m_*) and fuzzy relative importance ratings (${\tilde{\alpha }}_{m}$), competitive priority ratings (*β_m_*), and improvement ratios (*γ_m_*). Results show that visible dried fruit (*W*_3_) was the most important customer need (Table 4). This reflected that customer needs should be emphasized based on current product performance and goal. Table 5 shows the difference between relative importance ratings and final importance ratings.

**Table 4:** Goals and improvement ratio for customer needs (WHATs)

WHATs (*W_m_*)	Customer comparison matrix *X* = [*x_mn_*]_19 × 4_				Goals for WHATs (*g_m_*)	Improvement ratios for WHATs (*γ_m_* = *g_m_*/*x_m_*_l_)
	*B*_1_	*B*_2_	*B*_3_	*B*_4_		
*W*_1_	6.3	6.2	5.8	6.2	7	1.1053
*W*_2_	7.5	6.6	7.0	6.1	8	1.0640
*W*_3_	4.9	4.1	7.9	6.4	8	1.6489
*W*_4_	6.8	3.1	4.9	6.9	7	1.0328
*W*_5_	8.0	7.9	8.0	8.1	9	1.1198
*W*_6_	4.9	4.2	4.3	4.9	5	1.0150
*W*_7_	4.6	3.2	4.0	5.4	6	1.3171
*W*_8_	5.2	5.4	5.1	4.4	6	1.1489
*W*_9_	4.8	4.9	4.5	4.7	5	1.0465
*W*_10_	4.2	3.7	3.7	4.3	5	1.1947
*W*_11_	5.9	4.7	4.8	5.8	6	1.0189
*W*_12_	5.7	5.0	4.8	5.4	6	1.0452
*W*_13_	5.7	4.0	3.7	4.1	6	1.0588
*W*_14_	6.3	5.5	6.3	5.0	7	1.1183
*W*_15_	5.8	2.8	4.2	6.7	7	1.2038
*W*_16_	5.5	5.8	5.0	4.7	6	1.0872
*W*_17_	6.2	5.7	5.7	5.4	7	1.1317
*W*_18_	5.9	4.3	6.2	5.7	7	1.1887
*W*_19_	6.3	3.4	4.3	7.1	8	1.2781

**Table 5:** Comparison of relative importance ratings and final importance ratings of HOWs

Ranking	Relative importance ratings			Final importance ratings		
	WHATs (*W_m_*)	Crisp (*α_m_*)	Fuzzy (${\tilde{\alpha }}_{m}$)	WHATs (*W_m_*)	Crisp (*δ_m_*)	Fuzzy (${\tilde{\delta }}_{m}$)
1	*W*_16_	8.8	[7.8, 9.8]	*W*_3_	0.6428	[0.5634, 0.7221]
2	*W*_17_	8.6	[7.6, 9.6]	*W*_19_	0.5545	[0.4894, 0.6217]
3	*W*_11_	8.6	[7.6, 9.6]	*W*_7_	0.5363	[0.4638, 0.6023]
4	*W*_19_	8.4	[7.4, 9.4]	*W*_17_	0.5195	[0.4565, 0.5766]
5	*W*_18_	8.4	[7.4, 9.4]	*W*_16_	0.5155	[0.4556, 0.5725]
6	*W*_2_	8.3	[7.3, 9.3]	*W*_18_	0.5128	[0.4538, 0.5764]
7	*W*_4_	8.3	[7.3, 9.3]	*W*_15_	0.4930	[0.4308, 0.5539]
8	*W*_12_	8.2	[7.2, 9.2]	*W*_10_	0.4823	[0.4201, 0.5494]
9	*W*_14_	8.1	[7.1, 9.1]	*W*_14_	0.4785	[0.4172, 0.5347]
10	*W*_3_	8.1	[7.1, 9.1]	*W*_13_	0.4749	[0.4186, 0.5365]
11	*W*_6_	8.1	[7.1, 9.1]	*W*_11_	0.4659	[0.4126, 0.5211]
12	*W*_13_	8.1	[7.1, 9.1]	*W*_2_	0.4627	[0.4069, 0.5184]
13	*W*_15_	8.0	[7.0, 9.0]	*W*_12_	0.4575	[0.4035, 0.5155]
14	*W*_7_	7.7	[6.7, 8.7]	*W*_8_	0.4553	[0.3940, 0.5171]
15	*W*_10_	7.5	[6.5, 8.5]	*W*_6_	0.4413	[0.3868, 0.4958]
16	*W*_8_	7.4	[6.4, 8.4]	*W*_4_	0.4406	[0.3875, 0.4937]
17	*W*_5_	7.3	[6.3, 8.3]	*W*_5_	0.4204	[0.3628, 0.4780]
18	*W*_9_	7.1	[6.1, 8.2]	*W*_9_	0.3968	[0.3426, 0.4605]
19	*W*_1_	5.6	[4.6, 6.6]	*W*_1_	0.3262	[0.2680, 0.3845]

### 3.5 Technical attributes and sensory attributes (HOWs)

Thirty‐one technical and sensory attributes (HOWs) were generated based on literature review and expert interviews (Table 6). Sets of technical attributes consisted of required ingredients, and the processes of making oat granules and granola bars. The technical attributes (ingredients and process of making oat granule) were generated by consulting professionals with expertise in grain and oat granule manufacturing factories, respectively, while the attribute (process of making granola bar) was generated by literature review (Pathare, Baş, Fitzpatrick, Cronin, & Byrne, 2012). Sensory attributes were generated by this research.

**Table 6:** Technical attributes and sensory attributes (HOWs) according to customer needs (WHATs)

Technical attributes	Sensory attributes
Ingredients	Appearance
*H*_1_: amount of oat	*H*_12_: oat granule
*H*_2_: amount of other grains	*H*_13_: dried fruit
*H*_3_: amount of dried fruit	*H*_14_: nut
*H*_4_: amount of nut	*H*_15_: oat color
*H*_5_: amount of honey	*H*_16_: bar shape
*H*_6_: amount of sugar	Texture
Process of making oat granule	*H*_17_: crispiness
*H*_7_: nozzle air pressure	Aroma
*H*_8_: binder spray rate	*H*_18_: honey flavor
Process of making granola bar	*H*_19_: fruit flavor
*H*_9_: intensity of extrusion	*H*_20_: sweet flavor
*H*_10_: ratio of binder and oat granule	*H*_21_: sour flavor
*H*_11_: temperature of baking	*H*_22_: nutty flavor
	*H*_23_: grain flavor
	*H*_24_: toasted flavor
	Taste
	*H*_25_: honey taste
	*H*_26_: fruit taste
	*H*_27_: sweet taste
	*H*_28_: sour taste
	*H*_29_: nutty taste
	*H*_30_: oat taste
	*H*_31_: grain taste

### 3.6 The relationships between WHATs and HOWs

The ingredients’ technical and sensory attributes were considered to have a strong relationship with corresponding customer needs (appearance, texture, aroma, and taste) (data not shown). For instance, the amount of oat (*H*_1_) and oat granule on appearance (*H*_12_) had strong relation with visible oat granule (*W*_2_); amount of dried fruit (*H*_3_), dried fruit on appearance (*H*_13_), and fruit odor (*H*_19_) had strong relation with visible dried fruit (*W*_3_) and smelt dried fruit odor (*W*_8_), whereas moderate relations were considered between nozzle air pressure (*H*_7_), intensity of extrusion (*H*_9_) and crunchiness (*W*_16_), binder spray rate (*H*_8_) and slightly sweetness (*W*_13_), and temperature of baking (*H*_11_) and grain odor (*W*_6_), toasted odor (*W*_10_).

### 3.7 The relative technical ratings of HOWs

The relative crisp (*ε_n_*) and fuzzy technical ratings (${\tilde{\varepsilon }}_{n}$) were computed by crisp (*δ_m_*) and fuzzy final importance ratings (${\tilde{\delta }}_{m}$), and crisp and fuzzy relation scores (Table 7). Results show that the amount of nut (*H*_4_) and amount of oat (*H*_1_) were the two most important attributes that required to be improved. Although visible dried fruit (*W*_3_) was the most important customer needs, edible nut (*W*_19_), nutty odor (*W*_7_), and edible crunchy oat granule (*W*_17_) were the next important customer needs, which were affected more by the amount of nut and oat. The following priority for the improvement technical and sensory attributes results:

$$\begin{array}{c} \left(H_{4},H_{1}\right)\gg \left(H_{12},H_{14},H_{3},H_{13}\right)\gg \left(H_{11},H_{29}\right)\gg \left(H_{2},H_{19},H_{10}\right)\\ \gg \left(H_{30},H_{23}\right)\gg \left(H_{5},H_{31}\right)\gg \left(H_{22},H_{27}\right)\gg \left(H_{20},H_{26},H_{21}\right)\\ \gg \left(H_{6},H_{8},H_{18},H_{17},H_{24}\right)\gg \left(H_{15},H_{7}\right)\gg \left(H_{28},H_{25},H_{9}\right)\gg H_{9}. \end{array}$$

**Table 7:** Relative technical ratings of HOWs

HOWs	Relative technical ratings	
	Crisp (*ε_n_*)	Fuzzy (${\tilde{\varepsilon }}_{n}$)
*H*_1_	35.1022	[22.6689, 49.7564]
*H*_2_	29.5848	[17.9223, 43.5771]
*H*_3_	30.8829	[19.0891, 45.0341]
*H*_4_	36.2826	[22.5559, 49.6302]
*H*_5_	26.9598	[15.6143, 40.7099]
*H*_6_	23.0069	[12.1771, 36.1517]
*H*_7_	21.0339	[10.5196, 33.8329]
*H*_8_	22.8588	[12.0566, 36.0102]
*H*_9_	18.5223	[8.2916, 31.0317]
*H*_10_	29.0743	[17.4643, 43.0255]
*H*_11_	30.5911	[18.7744, 44.7353]
*H*_12_	31.5764	[19.6597, 45.7831]
*H*_13_	30.8664	[19.0621, 45.0052]
*H*_14_	31.2446	[19.4291, 45.3617]
*H*_15_	21.0737	[10.3944, 34.0653]
*H*_16_	12.1594	[2.6605, 23.9667]
*H*_17_	22.8385	[12.1075, 35.8652]
*H*_18_	22.8559	[12.0205, 36.0879]
*H*_19_	29.2588	[17.6520, 43.2003]
*H*_20_	24.1433	[13.1713, 37.4954]
*H*_21_	23.5870	[12.6871, 36.7843]
*H*_22_	25.2160	[14.0985, 38.6247]
*H*_23_	27.4164	[16.0763, 41.0811]
*H*_24_	22.6659	[11.8895, 35.7758]
*H*_25_	19.3561	[8.9686, 32.1402]
*H*_26_	23.9825	[13.0300, 37.2798]
*H*_27_	25.0588	[13.9741, 38.5043]
*H*_28_	19.5214	[9.1340, 32.2119]
*H*_29_	30.0078	[18.3195, 43.9646]
*H*_30_	27.8146	[16.4041, 41.5054]
*H*_31_	26.6847	[15.4388, 40.2472]

## 4 DISCUSSION

In line with our findings, a 7‐step model is proposed, as a modification of the 9‐step model. Testing on granola bars showed that the relative technical ratings could reflect the majority and importance of customer needs. The 7‐step model has three major characteristics.

First, by allocating precise values to technical measures of priority rankings, the 7‐step model communicates the importance and urgency of each technical measure for further product development. Bevilacqua et al. (2012) and Djekic et al. (2017) have proposed this idea in their research too, but they used HoQ for rating different products instead of developing a new product. After determining priority rankings, a development team will have a clearer indication of how to develop a product.

Second, by applying competitive analysis, technical priority rankings reflect a more competitive direction for development. De Pelsmaeker et al. (2015) included competitive analysis in the HoQ, but only made use of it for identifying the effects of different production processes on customer needs regarding European filled chocolate. However, in this research competitive analysis is incorporated for more precise importance ratings of customer needs.

Third, linguistic biases can be minimized by applying fuzzy scale and fuzzy sets for survey data and computing. Bevilacqua et al. (2012) applied fuzzy QFD to address the issue of vagueness and subjectivity in verbal expressions for rating the quality of extra virgin oil. Their findings resemble the results of this study.

Finally, the 7‐step model is easier to apply than a classical QFD. Steps related to correlation matrix of customer needs and precise value of technical measures were eliminated, since those steps do not directly contribute to the computation of priority rankings and are usually related to confidential information that is difficult to acquire. The 7‐step model is not only more practical to operate, but it also more efficiently transfers technical priorities to research and development teams in the company.

## 5 IMPLICATIONS FOR THE FOOD INDUSTRY

A successful new product requires development that is valued by customers (Jacobsen et al., 2014), a short time to market (Benner, Geerts, et al., 2003a), proper internal communication between marketing and technology functions (Jacobsen et al., 2014), and quality (Dijksterhuis, 2016). Quality function deployment can well respond to these requirements, since it has the benefit of reaching a compromise between customer needs and production techniques (Chien & Su, 2003), thereby decreasing production costs and reducing the production time (Dursun & Karsak, 2013). Furthermore, the 7‐step model as a modification of the classical QFD method can be a practical tool for new product development in the food industry. In conclusion, the 7‐step model can be a solution to the problem of successful new product development in the food industry.

## 6 RESEARCH LIMITATION

The 7‐step model can efficiently translate customer needs into technical priorities and diminish the communication gap between marketing and product development teams, making it a practical tool that can meet the needs of product development in the food industry. However, whether it can be applied to more complex food products, for example, more complex production conditions or materials, requires further research since only a simple granola bar was examined in this research. Other studies also have focused only on simple food products such as butter cookies (Bech, Engelund, Juhl, Kristensen, & Poulsen, 1994); peas (Bech et al., 1997); tomato ketchup (Costa et al., 2000); gold kiwi fruit leather (Vatthanakul et al., 2010); Korean beef barbecue (Park, Ham, & Lee, 2012); extra virgin oil (Bevilacqua et al., 2012); European filled chocolates (De Pelsmaeker et al., 2015); organic jelly (Cardoso, Casarotto Filho, & Cauchick Miguel, 2015); and Portobello mushrooms (Djekic et al., 2017). Costa et al. (2000) have also proposed that QFD is more suitable for products that assemble individual components. Accordingly, we suggest examining the 7‐step model on a more complex food product in further research. Another research limitation is related to the quality aspect. The values and process parameters indicated in the matrices were not tested because this was no access to laboratories to perform the tests. The values shown are suggested to be reviewed when real‐life applications are employed.

## 7 CONCLUSION

A 7‐step model was developed by modifying the 9‐step model by Chan and Wu (2005) and combining the sensory attributes by Bech et al. (1997) through incorporating competitive analysis to acquire final importance ratings of customer needs, applying fuzzy logic in surveys and interviews and replacing absolute values of relative technical ratings by priority rankings. Testing the 7 steps on granola bars showed that the relative technical priority ratings reflected the majority and importance of customer needs by incorporating competitive analysis and that implementing fuzzy logic led to more objective results. It is suggested that the 7‐step model is a practical tool for new food product development. For generalizability, further studies should be undertaken on other kinds of food products.

## ETHICAL STATEMENT

This study does not involve any human testing.

## CONFLICT OF INTEREST

The authors declare that they do not have any conflict of interest.
