# Supplementary material for: Quality function deployment modified for the food industry: An example of a granola bar
Source: Food Sci Nutr. 2019 Apr 3;7(5):1746–53. doi: 10.1002/fsn3.1014 (PMC6526655; doi:10.1002/fsn3.1014)
Supplement: Supplementary file 1 [file FSN3-7-1746-s001.docx]

# Appendix 1. Interview protocol for identifying customer need.

1. Respondent profile:
2. Age: □20-24 □25-30 □31-34 □35-40 □41-44 □Above 45
3. Have you eaten granola bar before? □Yes, I have. □No, I have not. (Please skip following questions.)
4. How often do you eat granola bar?
5. Why do you want to eat granola bar?
6. Do you prefer any particular brand of granola bar? Which one(s) do you prefer?
7. Customer need:
8. What are the required features of a granola bar regarding its appearance?
9. What are the required features of a granola bar regarding its aroma?
10. What are the required features of a granola bar regarding its taste?
11. What are the required features of a granola bar regarding its mouthful?

# Appendix 2. Survey questionnaire for rating customer needs.

1. Respondent profile:
2. Age: □20-24 □25-30 □31-34 □35-40 □41-44 □above45
3. Have you eaten a granola bar before? □Yes, I have. □No, I have not. (Please skip following questions.)
4. Please rate the importance of the following sensory attributes for a good granola bar according to your experience. (1 = very low; 2 = low; 3 = moderate importance; 4 = high; 5 = very high):

(1). Oat color, (2). Visible oat parts, (3). Visible dried fruit, (4). Visible nut, (5). Easy-to-eat bar shape, (6). Visible grain (sesame and pumpkin seeds) parts, (7). Nutty flavor, (8). Dried fruit taste, (9). Honey flavor, (10). Toasted flavor, (11). Grain (sesame and pumpkin seeds) flavor, (12). Oat flavor, (13). Slightly sweetness, (14). Dried fruit taste, (15). Nutty taste, (16). Crunchiness, (17). Crunchy oat granule, (18). Dried fruit parts, (19). Visible nut parts

# Appendix 3. Survey questionnaire for competitive analysis.

1. Respondent profile :
2. Age: □20-24 □25-30 □31-34 □35-40 □41-44 □above45
3. Have you eated a granola bar before? □Yes, I have. □No, I have not. (Please skip following questions.)
4. Evaluation on following four brands of granola bars (Brand A, Brand B, Brand C, Brand D) (1 = very poor; 2 = poor; 3 = neutral; 4 = good; 5 = very good)

(1). Oat color, (2). Visible oat parts, (3). Visible dried fruit, (4). Visible nut, (5). Easy-to-eat bar shape, (6). Visible grain (sesame and pumpkin seeds) parts, (7). Nutty flavor, (8). Dried fruit taste, (9). Honey flavor, (10). Toasted flavor, (11). Grain (sesame and pumpkin seeds) flavor, (12). Oat flavor, (13). Slightly sweetness, (14). Dried fruit taste, (15). Nutty taste, (16). Crunchiness, (17). Crunchy oat granule, (18). Dried fruit parts, (19). Visible nut parts

# Appendix 4. Survey questionnaire for determining relationship between customer needs and technical and sensory attributes.

Relation scores: 1= very weak; 2= weak; 3= moderate relation; 4= strong; 5= very strong.

*Technical attributes-Ingredients:* Amount of oat, Amount of other grains, Amount of dried fruit, Amount of nut, Amount of honey, Amount of sugar

*Technical attributes-Process of making oat granule bar:* Nozzle air pressure, Binder spray rate, Intensity of extrusion, Ration of binder and oat granule, Temperature of baking

*Customer needs* (1). Oat color, (2). Visible oat parts, (3). Visible dried fruit, (4). Visible nut, (5). Easy-to-eat bar shape, (6). Visible grain (sesame and pumpkin seeds) parts, (7). Nutty flavor, (8). Dried fruit taste, (9). Honey flavor, (10). Toasted flavor, (11). Grain (sesame and pumpkin seeds) flavor, (12). Oat flavor, (13). Slightly sweetness, (14). Dried fruit taste, (15). Nutty taste, (16). Crunchiness, (17). Crunchy oat granule, (18). Dried fruit parts, (19). Visible nut parts
